# MIND model for triple-negative breast cancer in syngeneic mice for quick and sequential progression analysis of lung metastasis

**DOI:** 10.1371/journal.pone.0198143

**Published:** 2018-05-29

**Authors:** Arnab Ghosh, Sandipto Sarkar, Snigdha Banerjee, Fariba Behbod, Ossama Tawfik, Douglas McGregor, Stephanie Graff, Sushanta K. Banerjee

**Affiliations:** 1 Cancer Research Unit, VA Medical Center, Kansas City, Missouri, United States of America; 2 Department of Anatomy and Cell Biology, University of Kansas Medical Center, Kansas City, Kansas, United States of America; 3 Department of Pathology and Laboratory Medicine, University of Kansas Medical Center, Kansas City, Kansas, United States of America; 4 Saint Luke’s Hospital of Kansas City, Kansas City, Missouri, United States of America; 5 Pathology Department, VA Medical Center, Kansas City, Missouri, United States of America; 6 Sarah Cannon Cancer Center at HCA Midwest Health, Overland Park, Kansas, United States of America; University of South Alabama Mitchell Cancer Institute, UNITED STATES

## Abstract

Mouse models of breast cancer with specific molecular subtypes (e.g., ER or HER2 positive) in an immunocompetent or an immunocompromised environment significantly contribute to our understanding of cancer biology, despite some limitations, and they give insight into targeted therapies. However, an ideal triple-negative breast cancer (TNBC) mouse model is lacking. What has been missing in the TNBC mouse model is a sequential progression of the disease in an essential native microenvironment. This notion inspired us to develop a TNBC-model in syngeneic mice using a mammary intraductal (MIND) method. To achieve this goal, Mvt-1and 4T1 TNBC mouse cell lines were injected into the mammary ducts via nipples of FVB/N mice and BALB/c wild-type immunocompetent mice, respectively. We established that the TNBC-MIND model in syngeneic mice could epitomize all breast cancer progression stages and metastasis into the lungs via lymphatic or hematogenous dissemination within four weeks. Collectively, the syngeneic mouse-TNBC-MIND model may serve as a unique platform for further investigation of the underlying mechanisms of TNBC growth and therapies.

## Introduction

Breast cancer is a genetically heterogeneous disease; it is the most frequently diagnosed and the second leading cause of cancer-related deaths in women aged 29–59 in the United States and globally[1–4]. Current therapies for breast cancer are potentially useful in improving patient survival. However, one-third of patients with aggressive triple-negative breast cancer (TNBC), representing 17–20 percent of all breast cancers [5–7], may relapse more frequently compared to receptor-positive subtypes [i.e., estrogen receptor (ER), progesterone receptor (PR), or human epidermal growth factor receptor 2 (HER-2)]. These 17–20 percent of TNBC patients eventually develop a distant metastatic disease, resulting in the patient’s death[5, 8–10]. Decades of studies help us understand the problem, but the underlying mechanisms of the pathobiology of breast cancer progression are still a mystery, and thus, a solution has yet to be found. Therefore, we are challenged to identify and understand the mechanism that drives breast cancer growth and progression, learn how to stop it, understand why some breast cancers become metastatic, and how to eliminate mortality associated with metastatic breast cancer. To precisely understand all these issues, a systematic study is required using a unique syngeneic animal model. Unfortunately, no such tractable *in vivo* model system is available to systematically study the metastasis progression of TNBC cells[11, 12].

Generation of an ideal tumor microenvironment that mimics a human tumor is challenging, and there are bottlenecking limits to it at multiple levels. [11, 13]. Mouse models with genetic alterations closely mimic the human tumor microenvironment and allow for studying the effect of one gene or a group of genes and their role in cancer progression and metastasis[11, 14–16]. Genetically engineered mouse models (GEMMs) for breast cancer research utilize a mammary-gland-specific promoter, such as mouse mammary tumor virus (MMTV) or whey acidic protein (WAP), that restricts the expression of the target gene in the epithelium of the mammary gland [17, 18]. GEMMs are frequently used to investigate the role of tumor-associated genes and their role in cancer progression and metastasis [11]. The added advantages of GEMMs, specifically, the MMTV promoter and Cre/loxP-mediated tumor suppressor gene deletion, are that they do not result in embryonic lethality[19]. In GEMMs, antibiotic (e.g., doxycycline) -mediated gene deletion or activation by an inducible system allows for conducting experimental manipulation of multiple genes for functional studies of tumor suppressor genes or oncogenes[20]. For example, our recent studies have shown that, by generating and utilizing a CCN5-conditional transgenic mouse model, CCN5 has restored ER-α expression and activity in mouse mammary epithelial cells, and suggest a novel mechanism of ER-α in breast epithelial cells[21]. However, most GEMMs, regardless of the degree of sophistication, tissue-specificity, intact immune system, or ability to mirror many relevant pathophysiological features of human cancer[19], involve a time-consuming process and are expensive with low experimental output[11].

Monitoring breast cancer tumor growth is possible by implanting immortalized cell lines or patient-derived tumor xenograft (PDTX) tissues subcutaneously or orthotopically into immunocompromised mice[11, 22, 23]. These models have several strengths but many weaknesses, including failure to incorporate the impact of the immune system[11, 24]. Besides lacking an immune system, PDTX modeling is an expensive, labor-intensive, and technically challenging procedure. To overcome the limitations of immunocompromised xenograft breast cancer models, an immunocompetent breast cancer mouse model has been introduced and is routinely used. In these models, mouse mammary cancer cell lines are implanted subcutaneously or into the mammary fat-pad of species-specific, syngeneic, immunocompetent mice for studies of rapid tumor growth and metastasis to the lungs, liver, and brain[11, 25–29]. However, this model does not display the sequential pathobiological changes in disease progression and metastasis, both of which are urgently needed for understanding the mechanism and validation of the efficacy of needed new drugs[30, 31].

The first available report of human intraductal injections of contrasting agents to be used for mammograms was published in 1972[32]. Since then, it has become a preferred route for drug screening and diagnostic purposes. Utilizing this form of injection, the mouse mammary intraductal (MIND) modeling of breast cancer was developed[33, 34], and then validated by others[35], and now it represents a clinically relevant model for ER-α-positive breast cancer [35]. Moreover, MIND is an ideal model for sequential studies of breast cancer progression from ductal carcinoma *in situ (*DCIS) to invasive cancer (IC), which then leads to hematogenous dissemination for metastasis [33, 35].

Although the MIND is a promising model and encompasses the biological diversity of human breast cancer, it is not yet clear whether this model can be used to study TNBC growth and progression in a syngeneic mouse. Thus, our goal is to develop a metastasis-TNBC MIND model in different syngeneic mice.

## Materials and methods

### Chemicals and antibodies

Dulbecco’s modified Eagle’s medium (DMEM), penicillin, streptomycin, Aprotinin, PMSF, Leupeptin, trypsin EDTA solution, and sodium pyruvate were purchased from Sigma Chemical Co. (St. Louis, MO, USA). Fetal Bovine Serum (FBS) was purchased from American Type Culture Collection (ATCC, Rockville, MD, Cat# 30–2020). Trypan blue was purchased from Stem cell Technologies (Cambridge, MA; cat #07050). Antibodies for Western blot analysis and immunohistochemistry were purchased from the following vendors: Anti-ER-α (Abcam, Cambridge, MA; Cat#ab32063), PR (Santa-Cruz, Dallas, TX; Cat#sc-166169), HER2 (Cell Signaling, Cat#4290), PCNA (Santa-Cruz, Dallas, TX; Cat#sc-56), α-smooth muscle actin (Santa-Cruz, Dallas, TX; Cat#sc-53142), Cytokeratin 19 (Santa-Cruz, Dallas, TX; Cat#sc-376126), β-catenin (Santa-Cruz, Dallas, TX; sc-7963), Twist(Abcam, Cambridge, MA; Cat#ab50581), Vimentin (Cell Signaling, MA; Cat#5741), Ep-CAM (Santa-Cruz, Dallas, TX, sc-25308), c-Myc Antibody (Santa-Cruz, Dallas, TX, sc-40) and CD44 (Santa-Cruz, Dallas, TX, sc-9960). Xylazine (100mg/ml) was purchased from (Sigma Aldrich, St. Louis, MO; cat#X1126). Ketoprofen (100mg/ml) and Ketamine (100mg/ml) were obtained from KCVA Pharmacy. The authentication certificates for all these chemicals, drugs, and antibodies were provided by these companies. Fresh working solutions of the chemicals were prepared once a month to guarantee effectivity.

### Cell lines and culture conditions

Mouse mammary tumor cell line Mvt-1, which was derived from a primary mammary tumor of a MMTV-VEGF/Myc bitransgenic mouse [36], was obtained as a gift from Dr. Danny Welch (KUMC) after written permission from Dr. Kent Hunter (NIH). The 4T1 mouse mammary tumor cell line, a TNBC type with highly metastatic cells derived from a spontaneously arising BALB/c mammary tumor [25], was obtained from American Type Culture Collection (ATCC, Rockville, MD, Cat# ATCC^®^ CRL-2539^™^). Cells were cultured in a monolayer in DMEM supplemented with 10% FBS, 2 mM glutamine, 100 unit/ml penicillin, and 100 unit/ml streptomycin in a 37°Cincubator in the presence of 5% CO_2_. Cells were used between four to six passes for any experiment and were checked every two months for mycoplasma contamination.

### Equipment and utilities

Instruments for mouse mammary gland intraductal injections were procured from the following vendors: 100μl capacity Hamilton syringe, with 26-gauge, 0.5-inch, 30^o-^angle, blunt-ended needle (Hamilton, cat # 80608); 1-ml BD slip-tip tuberculin syringe with 26.5 gauge needle (Thermos Fisher Scientific, Waltham, MA; cat # 309659); small scissors (Fine Science Tools, Foster City, CA; cat# 14028–10); Bonn artery scissors with a ball tip (Fine Science Tools, Foster City, CA; cat# 14086–09); micro-serrated Vannas spring scissors (Fine Science Tools, Foster City, CA; cat# 15007–08); Moria ultra fine forceps (Fine Science Tools, Foster City, CA; cat# 11370–40); Dumont #5—fine forceps (Fine Science Tools, Foster City, CA; cat# 11254–20); Dumont #7—fine forceps (Fine Science Tools, Foster City, CA; cat# 11274–20); delicate suture tying forceps (Fine Science Tools, Foster City, CA; cat# 11063–07); tissue forceps with 1x2 teeth (Fine Science Tools, Foster City, CA; cat# 11021–12); an auto clip system for wound closure with a 9mm clip applier (Fine Science Tools, Foster City, CA; cat# 12020–09); clip remover (Fine Science Tools, Foster City, CA; cat# 12023–00); and clips (Fine Science Tools, Foster City, CA; cat# 12022–09). As a part of post-operative care, an XpressHeat Pad (Sunbeam, Boca Raton, FL; cat# 002013-912-000) was used to maintain body temperature in the mouse.

### Human breast cancer tissue samples

The de-identified human breast cancer tissue samples were obtained from the tissue bank of University of Kansas Medical Center. The studies were conducted in accordance with the ethical guidelines approved by the Institutional Review Board of the University of Kansas Medical Center and VA Medical Center

### Animals

Female SCID mice were purchased from the Jackson Laboratory (Bar harbor, ME), female FVB/N mice and BALB/c wild-type mice were purchased from Charles River Laboratories (Wilmington, MA). All the animals used in this study were 8–12 weeks old. At least five mice per experiment were used for this study. Mice were housed in the Kansas City Veteran Administration Medical Center (KCVAMC) animal care facility. Mice were fed commercial mouse diet food (Envigo, Indianapolis, IN) with a 12-hour light-dark cycle under pathogen-free conditions. Experimental animals were carefully monitored throughout the course of the experiment and if they showed signs of distress including, but not limited to, reduced food or water intake, reduced activities, hunched posture, weight loss, vocalization, irritability or lack of grooming, they were euthanized. Euthanasia was performed using CO2 inhalation followed by cervical dislocation.

### Ethics statement

Animal protocols were approved by the KCVAMC animal care and use committee, according to the AAALAC guidelines and NIH guidelines for the care and use of laboratory animals. Mice were under continuous daily observation pre-and post-operation. All animals were treated humanely, and they were euthanized as per required experimental time point or if they displayed excessive discomfort, whichever was earlier.

### Mouse mammary intraductal (MIND) method

**Fig 1:**
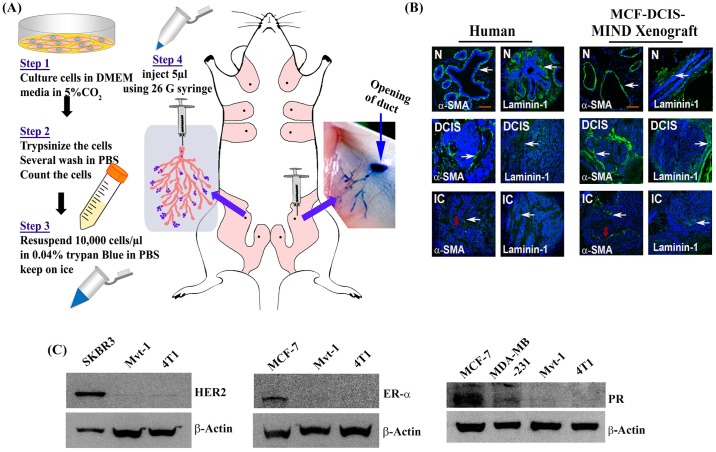
Validation of MIND model and detection of different receptors’ status in various human and mouse breast cancer cell lines. **(A)** Schematic representation of mouse mammary intraductal injection through cleaved nipples. Mvt-1 or 4T1 cells were re-suspended in 0.04% trypan blue and injected through 4^th^ inguinal mammary gland cleaved nipple. The experimental protocols are depicted in the left two panels. **(B)** Representative images of α-SMA and laminin-1 immunostaining in human biopsy samples (n = 26) and MCF-DCIS-MIND xenograft samples (n = 5). The images illustrate the similarity of the Human breast and MIND xenograft architecture. White arrows indicate staining (green) of laminin and α-SMA and Red arrows indicate possible localized death of MECs. Scale bar, 50μm. **(C)** Representative Western blot analysis of ER-α, PR and HER2 expressions in the cellular extracts of SKBR3, MCF-7, MDA-MB-231, Mvt-1, and 4T1 cell lines. SKBR3 cell extract was used as positive control for HER2, and MCF-7 cell extract was used as positive control for ER-α and PR. (n = three replicates). All photographs are cropped from original figures.

The procedure of the MIND model is summarized in Fig 1A. Briefly, human breast cancer cells (MCF-DCIS) and mouse mammary cells (Mvt-1 or 4T1) were suspended in 0.04% trypan blue in PBS. The concentration of cells was 10,000 cells/μl. The final volume did not exceed five μl per injection per gland. The mouse was sedated for one hour with an intraperitoneal injection of Ketamine 1μg/g body weight and Xylazine 35μg/g body weight, using a 1 ml tuberculin syringe [37]. Ketoprofen at 5μg/g body weight was injected as an analgesic and antipyretic agent [34]. After complete sedation, the fur coat on the lower abdomen adjacent to the inguinal nipples was removed using hair-removing lotion (Nair Hair Remover Lotion; cat# B001G7PTWU). The mouse was placed on a surgery platform, and the area adjacent to the inguinal glands were cleaned (70% alcohol) and prepared for surgery. Spring scissors were used to cut the nipple at the base while holding the nipple with an ultra-fine force. Optimum duct access was achieved on both the inguinal mammary glands. Using small scissors and ball-tip scissors, a longitudinal inverted Y-shaped incision was made in the abdomen slightly below the inguinal nipple. Cuts were made between 4^th^ and 5^th^ nipples on both sides [33, 34]. The inguinal glands were exposed by separating the skin using fine forceps. The cells were injected with a Hamilton syringe through the nipple, holding the syringe in the same direction of the duct. After successful injection, any visible cell residue around the duct was wiped out. The incision was closed with the auto-clip system. The mouse was placed on a heating pad for recovery to compensate a loss of body temperature. Ketoprofen (5μg/g body weight) was injected intraperitoneally 24 hours after the operation. Post-operation, mice were fed the same commercial mouse diet and were under continuous observation. Seven days after the operation the clips were removed. Mice were monitored every other day for any sign of tumor growth, and once the tumors were palpable, tumor volume was measured three times per week using an electronic caliper with study-log software (Studylog Systems, Inc. South San Francisco, CA).

### Whole mount staining of mouse mammary glands

Mouse 4^th^ inguinal mammary glands were harvested at different time points and spread on a poly-l-lysine coated microscope slide (Fisher Scientific, Hampton, NH). The gland was subjected to whole-mount fixation, defatting and stained with carmine. Briefly, after glands were adhered to the slide (1–2 min), they were fixed in Carnoy’s fixative [100% ethanol and 30% acetic acid (3:1)] for 4h at room temperature. The slides were washed in 70% ethanol for 30 min, then in distilled water. The glands were stained with carmine stain [1 g Carmine (Sigma-Aldrich C-6125) and 2.5 g aluminum potassium sulfate (Sigma-Aldrich C-237,086) in 500 ml water] overnight in the dark. The tissues were washed in 70, 90, and 100% ethanol. Tissues were cleared in xylene, and a large cover glass was used to mount the gland with per-mount for long-term storage [38].

### Western blot analysis

The Western blot assay was same as described previously [39]. Briefly, cells were washed with PBS and lysed in RIPA buffer. An equal amount of cell lysate proteins was subjected to 10% SDS-PAGE, followed by Western blot analysis. The gel images were detected with Super Signal Ultra Chemiluminescent Substrate (Thermo Fisher Scientific, Waltham, MA) and captured using Kodak one dimensional image analysis software, Version 3.6 (Carestream, Rochester, NY). Original pictures of the Western blot were cropped and organized in Fig 1.

### Immuno-staining

Mouse lungs were fixed with intratracheal instillation of fixatives [40], and immunohistochemical and immunofluorescence analysis was performed per the previous method [41]. Briefly, 5μm-thick tissue sections were deparaffinized in Xylene, followed by gradual rehydration in grades of alcohol, repeatedly washed in PBS, and non-specific antibody binding was blocked with a ready-to-use blocking solution (Thermo-Fisher Scientific, Waltham, MA; cat# 50062Z). Primary antibodies were incubated overnight at 4°C followed by incubation with specific secondary antibodies. DAB staining was performed for immunohistochemical detection. The sections were imaged using a Leica photomicroscope. The clinical stages were confirmed by a pathologist using adjacent hematoxylin and eosin stained slides.

### In vivo imaging

Mvt-1 cells were stably transfected with the pZsGreen1C1 construct (Takara Bio USA; Cat # 632447) using EndoFectinTM Max Transfection Reagent (GeneCopoeia; Cat # EFM1004-01-S). Transfected cells were injected into the 4th inguinal mammary ducts of FVB/N mice as described in an earlier section. The mice were anesthetized and imaged at one-week intervals up to 21days using Bruker In-vivo F-Pro imaging system.

### Statistics

The statistical analysis was performed using the Graph Pad Prism 6 software and PASS^15^ software. All data are expressed as the mean ± SD. Statistically significant differences between groups were determined by using an Anova analysis with Bonferroni post hoc test.A value of P < 0.05 was considered statistically significant.

## Results

### Development and characterization of triple negative MIND tumor xenograft with metastasis in syngeneic mouse model

The MIND model, in which breast cancer cells are injected through the mammary duct via nipple, was initially developed by Behbod *et*.*al*. [33], and this model is now considered to be an ideal pre-clinical model [35]. In this study, our initial goal was to validate the importance of this model for preclinical studies. To do so, we generated a MIND model by injecting MCF-DCIS (MCF-DCIS.com) cells into the mammary duct of immunodeficient SCID mice as depicted in Fig 1A and compared the morphology of normal, DCIS and disease progression in MCF-DCIS-MIND xenograft and human tissue sections. The ductal network in mouse mammary gland and human breast is composed of two essential layers of cells: luminal epithelial (LE) and myoepithelial cells (MECs) [42–47]. MECs are localized between the LE cells and stroma [43, 48–51]. MECs play a key role in the organization and development of mammary glands, act as a “biological fence,” and help in maintaining tissue integrity of the breast [42]. MECs contribute significantly to basement membrane (BM) production by enhancing the expression and deposition of BM components such as Laminin-1 and Collagen IV, which are required for ductal organization [42, 52]. MECs are present in the normal, premalignant breast as well as in DCIS [48]. To spread, cancer cells change the way they operate in DCIS, and as a result, the growth of MECs is hindered and gradually disappears [48, 53, 54]. Since MECs prevent initial phases of breast cancer development, such as DCIS-invasion (IC) transition, MECs have been considered natural tumor suppressors and an ideal prognostic marker for DCIS to IC progression [55, 56]. Given the importance of MECs in breast cancer progression, we determined and compared the status of Laminin 1 and α-SMA (a MEC marker) in human breast cancer sections with various stages including adjacent normal and mouse mammary tumor sections obtained from a MCF-DCIS-MIND xenograft model. Consistent with the staining patterns of human samples, we found duct-like structures, which were surrounded by Laminin 1 and contained a layer of cells positive for α-SMA (Fig 1B). Analysis of the tumors at different time points (3–8 weeks) revealed a progression from DCIS to invasive phenotypes. In DCIS, both α-SMA and Laminin-1 were expressed in the myoepithelial cells (MECs). This layer disappeared as tumors became invasive (Fig 1B). Collectively, this study suggests that the MIND model is a surrogate model for the pre-clinical studies of breast cancer pathophysiology.

Our subsequent goal was to develop and characterize a TNBC-MIND model in syngeneic mice. There are several mouse mammary tumor cell lines available, and many of them have divergent metastatic behavior in the syngeneic mouse models [57, 58]. Among the available mouse cell lines, two highly metastatic TNBC cell lines were considered for these studies. These include Mvt-1 and 4T1 breast cancer cell lines [25, 59–62]. Based on their origin, these two cell lines are considered TNBC cell lines. We further validated the triple-negative behavior of these two cell lines by characterizing the common hormone receptor status by Western blot analysis using specific antibodies that react with both humans and mice. The studies revealed that the expressions of ER-α, PR and HER2 were undetected in both Mvt-1 and 4T1cell lines, further confirming the triple-negative status of these two cell lines (Fig 1C). The cell lysates of MCF-7 and SKBR3 were used as positive controls for ER, PR, and HER2 receptors, while the MDA-MB-231 cell lysate was used as negative control for all receptors.

The 4T1 cell line and 4T1 cell generated tumor xenograft are well-characterized by various laboratories, and the authentication of this cell line was confirmed by the vendor (ATCC). Thus, further verification is not necessary for this study. However, the Mvt-1 cell line is comparatively less characterized, and thus a further validation is required. Since c-Myc would be the best possible marker to identify the Mvt-1 cells as this cell line is generated from a MMTV-VEGF/Myc bitransgenic mouse, we sought to determine the status of c-Myc in the Mvt-1 cell line for authentication. We successfully corroborated the cell line as Mvt-1 for further studies by detecting c-Myc expression using immunofluorescence analysis (S1A–S1C Fig).

Finally, we explored the tumor-forming ability of Mvt-1 and 4T1 cells in the mammary ducts of female immunocompetent FVB/N and BALB/c mice, respectively. To do so, Mvt-1 or 4T1 cells [10,000 cells/μl in phosphate-buffer saline (PBS)] were injected into the ducts of the 4th inguinal mammary glands through the nipple of female mice, as described in Fig 1A using brief modifications of the previous method [33]. We found that both cell lines formed intra-ductal tumors in these mice, and the tumor was palpable within two weeks after the injection of the cells (S2 Fig).

### Evaluation and characterization of the development of primary tumors and lung metastasis in the Mvt-1-MIND tumor xenograft model

**Fig 2:**
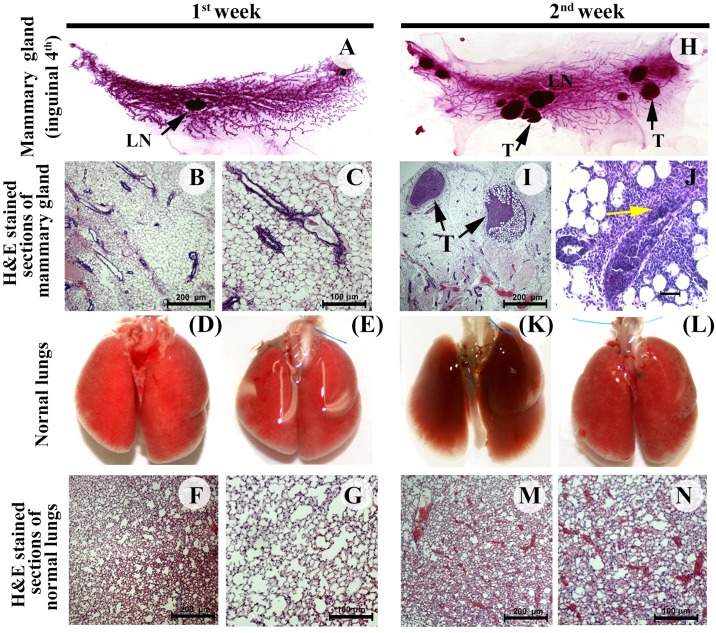
Development of primary tumors in the mammary ducts of female FVB/N mice following Mvt-1 cells injection through MIND method. **(A)** Representative photograph of whole mount of 4^th^ inguinal mammary glands of female mice (FVB/N) injected with Mvt-1 cells for one week (n = 5). LN (arrow); Lymph node. **(B-C)** Representative H&E staining in serial sections of the mammary glands of female FVB/N mice injected with Mvt-1 cells for one week (n = 5). Images illustrate normal ducts and lobules (B-C) in the mammary glands. Scale bars represent 100–200 μm. **(D-E)** Representative photograph of lungs of FVB/N mice injected with Mvt-1 cell for one week (n = 5). The images illustrate no outgrowth of metastasis in the lungs. **(F-G)** Representative H&E staining serial sections of lungs of female FVB/N mice injected with Mvt-1 cells for one week (n = 5). The images show no detectable microscopic metastasis in the lungs. Scale bars, 100–200 μm. **(H)** Representative whole mount of 4th inguinal mammary gland of female FVB/N mice injected with Mvt-1 cells for two weeks (n = 5). Image shows palpable tumors in mammary gland. Arrow (T) indicates tumor, LN; Lymph node. **(I-J)** Representative H&E staining sections of mammary glands collected from the female FVB/N mice injected with Mvt-1 cells for two weeks (n = 5). The photographs illustrate DCIS-like structure (I) and micro-invasion (J).; T; Tumor. Black arrows indicate DCIS-like structures, and yellow arrow indicates microinvasion. Scale bar, 100–200μm. **(K-L)** Representative photograph of lungs of female FVB/N mice injected with Mvt-1 cell for two weeks (n = 5). The images illustrate no metastatic outgrowth in the lungs. **(M-N)** Representative H&E staining serial sections of lungs of female FVB/N mice injected with Mvt-1 cells for two weeks (n = 5). The images show no detectable microscopic metastasis in the lungs. Scale bars, 100–200 μm.

The goal of this study was to examine the time-dependent progression of Mvt-1-tumor growth in the mammary ducts and distant organs of female FBV/N mice; we monitored primary tumor growth and metastasis based on a gross and histopathological analysis. The histopathology of the primary tumors and metastasis was examined by the pathologists (OT and DM). After the first week of intraductal injection of Mvt-1 cells, there were no visible primary tumors in the mammary glands (Fig 2A). Serial sections of the entire fourth inguinal mammary gland also revealed no apparent tumor mass upon hematoxylin and eosin (H&E) staining (Fig 2B and 2C), and cancer cells were undetected in the lungs, lymph nodes, or blood vessels (Fig 2D–2G and S3 Fig).

Palpable tumors appeared two weeks after cell injection in all mice (n = 5), and the growth of tumors was easily detected in the whole mount of the 4th inguinal mammary glands and H&E-stained tissue sections (Fig 2H and 2I). At this point, the structure of the ducts was still intact in most of the cases, but with only a few exceptions, the cancer cells invaded into the stroma (Fig 2J). No lung metastasis was observed macroscopically or microscopically (histology) (Fig 2K–2N).

**Fig 3:**
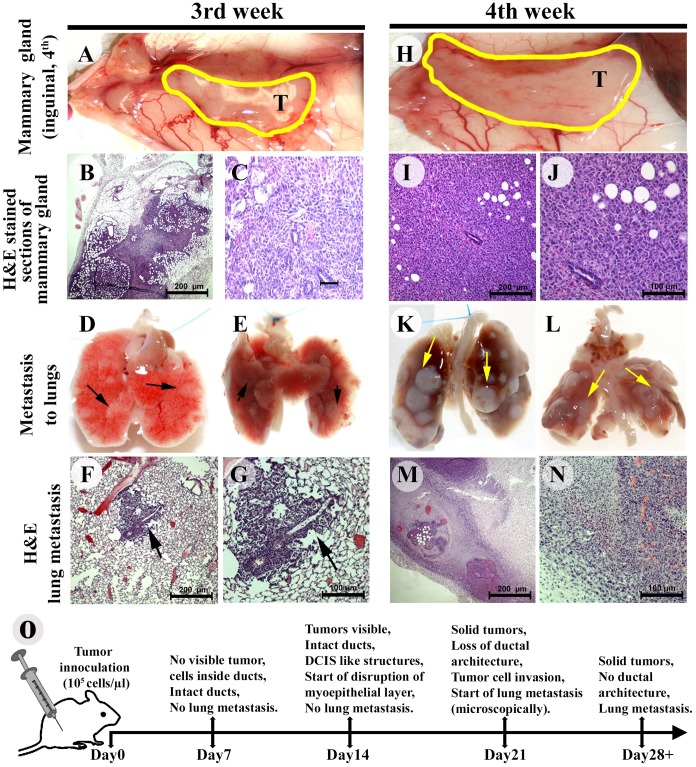
Primary mammary tumor growth, progression and outgrowth of metastasis in the lungs of female FVB/N mice following Mvt-1 cell injection through MIND method. **(A)** Representative photograph of the 4th inguinal mammary gland with tumor (T) of female mice (FVB/N) injected with Mvt-1 cells for three weeks (n = 5). The photograph illustrates tumor growth in the mammary ducts. **(B-C)** Representative H&E staining sections of the mammary gland of female FVB/N mice three weeks after the injection of Mvt-1 cells (n = 5). Images illustrate local invasion. Scale bars, 50–200 μm. **(D-E)** Representative photograph of lungs of female FVB/N mice injected with Mvt-1 cell for three weeks (n = 5). Photographs illustrate small outgrowth of metastasis (black) in the lungs (arrows). **(F-G)** Representative H&E staining sections of the lungs of female FVB/N mice injected with Mvt-1 cells for three weeks (n = 5). Arrows indicate microscopic metastatic growth in the lungs. Scale bars, 100–200 μm. **(H)** Representative photograph of the 4th inguinal mammary glands with tumor (T) of female mice (FVB/N) injected with Mvt-1 cells for four weeks (n = 5). **(I-J)** Representative H&E staining sections of the mammary gland of female FVB/N mice four weeks after the injection of Mvt-1 cells (n = 5). Images illustrate local invasion. Scale bars, 100–200 μm. **(K-L)** Representative photograph of lungs of female FVB/N mice injected with Mvt-1 cells for four weeks (n = 5). Photographs illustrate large outgrowths of metastasis (white) in the lungs (arrows). **(M-N)** Representative H&E staining lung sections with metastasis from female FVB/N mice injected with Mvt-1 cells after four weeks (n = 5). Scale bars, 100–200 μm. **(O)** The schematic diagram illustrates the summary of the duration of the sequential growth of lung metastasis from preneoplastic lesions following injection of Mvt-1 cells into the ducts through MIND method.

**Fig 4:**
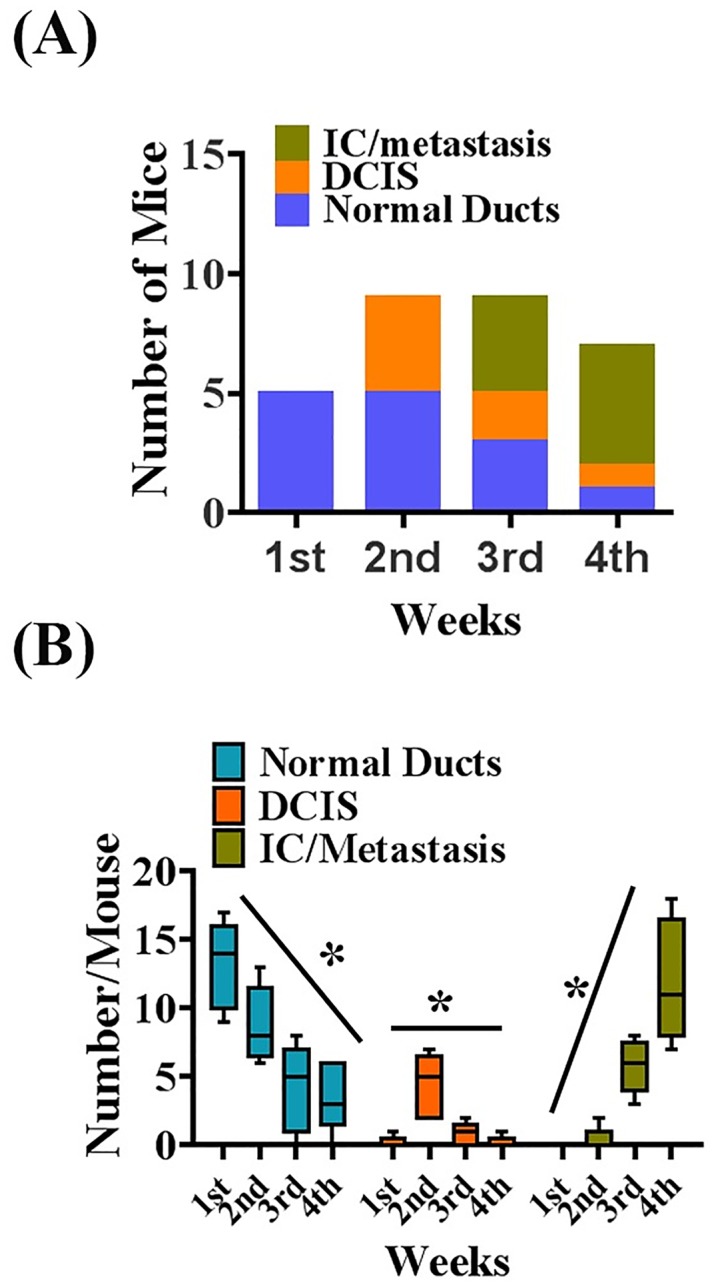
Quantitative evaluation of tumor development in FVB mouse fourth inguinal mammary gland injected with Mvt-1 cells. **(A)** Graph depicts the number of mice bearing normal ducts, DCIS and IC/Metastasis (n = 5). **(B)** Representation of the count of available normal mammary ducts, DCIS and IC/metastatic tumors in individual mice. (DCIS: Ductal carcinoma in-situ and IC: Invasive carcinoma). Data presents Mean ±SD. *indicates p<0.001 in an Anova analysis with Bonferroni post hoc test.

Primary tumor growth, local invasion, and metastasis to the lungs were observed in four out of five mice after the third week of cell injection (Fig 3A). The ducts containing cancer cells lost their morphology, and cancer cells invaded the surrounding ducts, lobules, and stroma (Fig 3B and 3C). Small metastatic outgrowths were detected in the lungs three weeks after injection (Fig 3D and 3E), and the cancer cells in the lungs were evident from the histology of the sections (Fig 3F and 3G). Four weeks after cancer cell injection, all the ducts were surrounded by dysplastic cancer cells, with very little intact ductal morphology (Fig 3H–3J). Moreover, we found that the volume of tumors per gland was increased in a time-dependent fashion (Fig 4) without any significant loss of body weight (S4 Fig).

Multiple large outgrowths of metastases in the lungs were detected at four weeks after the injection of Mvt-1 cells (Fig 3K–3N). In this study, no tumor cells were found in lymph nodes (S5 Fig). However, tumor cells were identified in large dilated blood vessels in mice with metastasis (S5 Fig). Collectively, this study indicated that gradual progressions of primary tumor growth and lung metastasis were achieved after three to four weeks after injection of Mvt-1 cells in the mammary ducts (Fig 3O). Moreover, we found that c-Myc is highly expressed in primary tumors and metastatic cells in the lungs (S1 Fig), indicating both primary tumors and metastasis are of Mvt-1 cell origin.

We, next, examined whether the contralateral glands of the mice were affected by tumor burden. We found that no primary tumors had developed in any contralateral glands where tumor cells had not been injected (S6 Fig).

**Fig 5:**
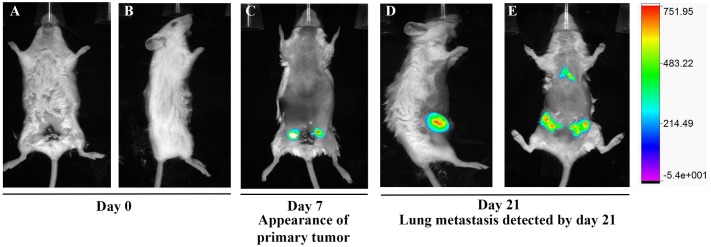
In vivo imaging of GFP-signal in tumor-bearing mice. **(A-E)** Representative whole-body imaging of female FVB/N mice after injecting GFP-labeled Mvt-1 cells. Mice were imaged at Day 0 showing no signal (A &B), day 7 exhibiting signal in the primary tumors (C) and Day 21 illustrating signals in both primary tumors and lung metastasis. (n = 5 mice). The right panel shows the scale of the intensity.

Finally, to further corroborate the tumor-forming and metastatic ability of Mvt-1 cells, GFP-labeled Mvt-1 cells were injected into the ducts through the nipples of the 4^th^ mammary glands of female mice, and tumor growth and metastasis in the lungs was monitored non-invasively using *in vivo* imaging system (Bruker). The studies showed that the fluorescence signal was first detected at day 7 in the 4^th^ mammary ducts of tumor-bearing mice. Then at day 21, signals were detected both in glands and lungs in the tumor-bearing mice with higher fluorescence signal in the glands as compared to the glands of day 7 (Fig 5). Collectively, these studies indicate that Mvt-1 cells have the potential to form primary tumor growth in the mammary glands as well as metastasis in the lungs.

### Validation of the sequential progression of mammary cancer from DCIS to local invasion and metastasis in the Mvt-1-MIND tumor model

**Fig 6:**
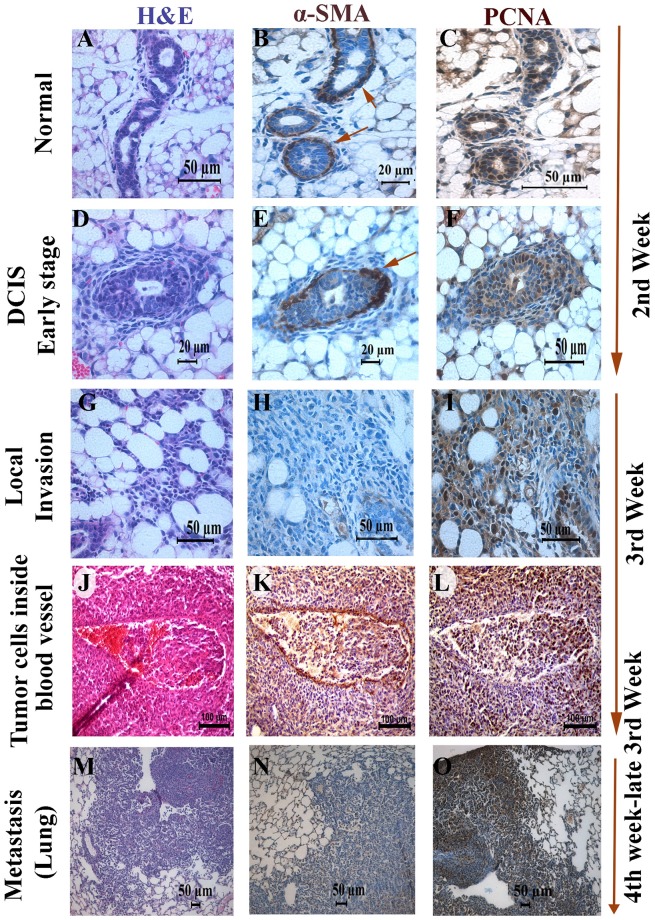
Immunohistochemical characterization of primary tumors and lung metastasis. **(A-C)** Representative photographs of H&E staining, α-SMA and PCNA immunohistochemical staining sections of normal ducts and lobules of the mammary gland of female FVB/N mouse injected with Mvt-1 cells for one week (n = 5). The ductal architecture demarcated by α-SMA (B) and the presence of Mvt-1 cells inside duct detected by PCNA immune-staining (C). Scale bar, 20–50 μm. Arrow indicates α-SMA-positive myoepithelial layer. **(D-F)** Representative photographs of H&E staining, α-SMA and PCNA immunohistochemical staining sections of an early stage of a DCIS-like structure formed in the mammary glands of a female FVB/N mouse injected with Mvt-1 cells for two weeks (n = 5). α-SMA immunostained the myoepithelial layer around the DCIS (E), and PCNA immunostained the highly proliferative Mvt-1 cells inside DCIS structure (F). Scale bar, 20–50 μm. Arrow indicates α-SMA-positive myoepithelial layer. **(G-I)** Representative photographs of H&E staining, α-SMA and PCNA immunohistochemical staining sections of an invasive area of the mammary glands of a female FVB/N mouse injected with Mvt-1 cells for three weeks (n = 5). Scale bar, 50μm. **(J-L)** Representative photographs of H&E staining, α-SMA and PCNA immunohistochemical staining in the blood vessels of a female FVB/N mouse injected with Mvt-1 cells for four weeks (n = 5). In this model, the preferred route of cell migration toward other organs to form a secondary tumor is via blood vessels. This staining shows a clear indication of cells delineating from adjacent tumors inside blood vessels. Scale bar,100 μm. **(M-O)** Representative photographs of H&E staining, α-SMA and PCNA immunohistochemical staining sections of lung metastasis of a female FVB/N mouse injected with Mvt-1 cells for four weeks (n = 5). Scale bar, 50μm.

To validate the sequential progression of breast cancer, we investigated the status of molecular markers of proliferation and invasion, such as a proliferating cell nuclear antigen (PCNA) [63] and α-SMA in different paraffin-embedded tissue sections using immunohistochemical analysis. The histology of each section was confirmed by H&E-staining (Fig 6A). In normal ducts, as expected, the outer α-SMA positive myoepithelial layer was intact (Fig 6B), and some of the normal ductal epithelial cells were PCNA positive, indicating that Mvt-1 cells were alive and in proliferation mode (Fig 6C).

DCIS-like structures were visible two weeks after Mvt-1 cell injection (Fig 6D). We found that the outer myoepithelial layer of the DCIS-like structure was α-SMA positive, and the Mvt-1 cells, which were located inside the ducts and formed DCIS-like structures, were PCNA positive, indicating Mvt-1 cells could form DCIS-like structures within a brief period after injection into the mammary ducts of FVB/N mice via proliferation (Fig 6E and 6F).

By the end of the second week or early third week, we observed that several Mvt-1 cells infiltrate the surrounding stroma of the mammary gland (Fig 6G–6I). These infiltrating cells were mostly PCNA positive (Fig 6G), suggesting that these infiltrating cells are in proliferation mode.

Next, we investigated whether the infiltrated PCNA overexpressing cells formed any metastatic growths in the lungs; the lungs are the most attractive soil for the secondary growth of breast cancer cells in a xenograft model [17]. By the end of the third week, Mvt-1 cells had formed secondary tumors in the lungs with elevated levels of PCNA expression (Fig 6M–6O), traveling through the blood vessels (Fig 6J–6L). The blood vessels were identified by immunohistochemical reactions of α-SMA (Fig 6K), an ideal marker for the identification of blood vessels [64, 65]. In this study, we found that once the proliferating cancer cells started to infiltrate, highly aggressive tumor cells began to intravasate and form embolisms in the bloodstream. The embolism facilitated the further accumulation of tumor cells in the blood vessels, and eventually extravasated to the lung to form a secondary growth.

### 4T1 cells mimic tumor progression and metastasis patterns in the same manner as Mvt-1 cells

**Fig 7:**
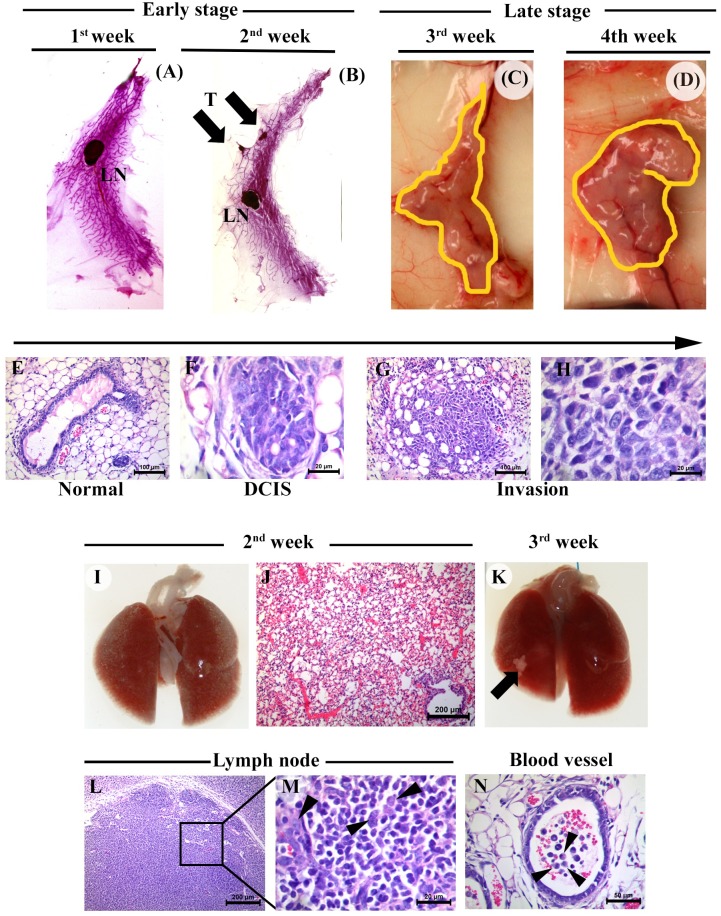
Tumor progression and metastasis in the mammary ducts of female BALB/c mice following injection of 4T1 cells through MIND method. **(A)** Representative photograph of the whole mount of 4^th^ inguinal mammary gland after the first week of 4T1 cell injection into the mammary ducts of female BALB/c wild-type mouse (n = 5). Note, no palpable tumors are apparent. LN, lymph node. **(B)** Representative photograph of the whole mount of 4^th^ inguinal mammary gland after the second week of 4T1 cell injection into the mammary ducts of female BALB/c wild-type mouse (n = 5). Note, tumors are palpable (arrow). T, tumor, and LN, lymph node. **(C-D)** Representative photograph of 4th inguinal mammary glands of female BALB/c mouse injected with 4T1 cells for three weeks and four weeks, respectively (n = 5). The photograph illustrates large tumor growth in the mammary ducts (yellow lines). **(E-H)** Representative photographs of H&E-staining sections illustrate the sequential progression of tumor growths from DCIS to the invasion in the mammary ducts of a female BALB/c mice injected with 4T1 cells (n = 5). Scale bar, 20–100 μm. **(I-K)** The representative photograph of lungs of 4T1-tumor-bearing female BALB/c mice illustrate no metastatic growth macroscopically (I) or microscopically (J) (n = 5). However, a small outgrowth in the lungs (arrow) was detected by the third week, indicating initiation of metastasis to lungs (K). Scale bar, 200 μm. **(L-N)** Representative H&E staining sections of lymph nodes (L and M) and blood vessels (N) illustrate intravasation of 4T1 cells after three weeks of injection (n = 5). Box indicates the enlarge margination of Fig L. Arrow heads indicate 4T1 cells in the lymph node and blood vessels. Scale bars, 20–200 μm.

Encouraged by our finding using Mvt-1 cells, we repeated the entire experimental setup using 4T1 cells in BALB/c mice. Results indicate that 4T1 cells are equally efficient in forming tumors in mouse mammary ducts within the same timeframe as observed in the Mvt-1-MIND model, starting with tumor initiation to DCIS, invasion, and finally metastasis to the lungs (Fig 7A–7K). Interestingly, we found that 4T1 cells, unlike Mvt-1, disseminate through the lymphatic system (Fig 7L and 7M), as well as through the blood vessels (Fig 7N) for colonization to the lungs, and finally metastatic outgrowth.

### Predictive role of mesenchymal properties in the progression of Mvt-1 tumor growth and metastasis in MIND model

**Fig 8:**
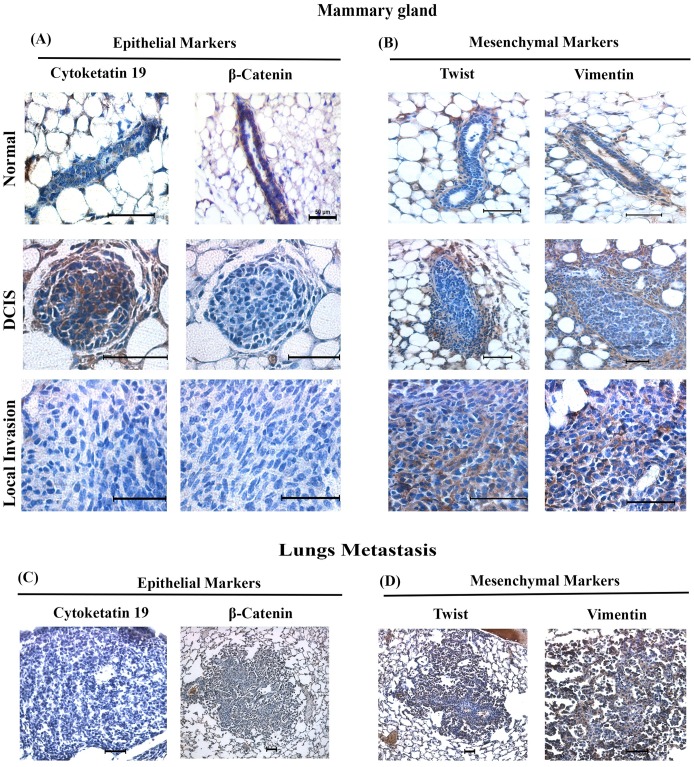
Detection of EMT markers in the non-invasive and invasive tumor in TNBC-MIND model. **(A)** Representative photographs of Cytokeratin 19 and β-catenin immunostaining serial sections of mammary glands of FVB/N mice injected with Mvt-1 cells for various times (n = 5). Scale bar, 25–200 μm. **(B)** Representative photographs of Twist and Vimentin immunostaining serial sections of mammary glands of female FVB/N mice injected with Mvt-1 cells for various times (n = 5). Scale bar, 20–200 μm. **(C)** Representative photographs of Cytokeratin 19 and β-catenin immunostaining sections of lungs illustrating no expression of epithelial markers in metastatic tumors generated by Mvt-1 cells in lungs (n = 5). Scale bar, 50–200 μm. (**D)** Representative photographs of Twist and Vimentin immunostaining sections of lungs illustrating elevated levels of mesenchymal markers in metastatic tumors generated by Mvt-1 cells in the lungs (n = 5). Scale bar, 100–200 μm.

The epithelial–mesenchymal transition (EMT) is a unique process that is essential for embryonic development. However, cancer epithelial cells, by sharing the EMT mechanism, acquire aggressive phenotypes including invasion and metastasis [66–68]. Given the aggressive behavior of Mvt-1 cells, we determined the status of epithelial-mesenchymal markers in Mvt-1-primary tumor xenografts and lung metastasis using immunohistochemical analysis. We found that mesenchymal markers such as Twist and vimentin were overexpressed in both primary tumors and lung metastatic sections as compared to epithelial markers (i.e., cytokeratin 19 and β-catenin), which were undetected or minimally detected in the DCIS-like structure, primary tumors, and metastatic samples (Fig 8). Further, the studies show that Twist and Vimentin were minimally expressed in the DCIS-like structures (Fig 8B), and thus suggesting Twist and Vimentin could be intimately involved in invasion and metastatic growth of Mvt-1 cells without interfering with the formation of DCIS-like structure in these cells. However, further studies are warranted to establish the role of EMT in this event.

## Discussion

The greatest number of TNBC patients die with metastatic disease [69] because currently available therapeutic options can cure only the well-confined primary tumors from which metastatic lesions arise [70]. Despite knowing the facts, the progression of the invasion-metastasis cascade from primary tumors is still unclear at the molecular level. Thus, it is urgently necessary to dissect the molecular mechanisms of the progression of the disease using appropriate models.

**Fig 9:**
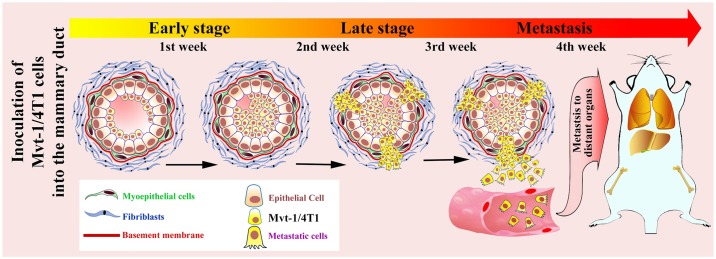
Schematic representation of the timescale of the progression of the TNBC from DCIS to metastatic growth in lungs in syngeneic mouse MIND model.

Multiple animal models have been developed recently, but these models are not designed appropriately to study the step-by-step progression of metastasis in TNBC [17, 69, 71]. Therefore, the aim of the present study was to develop a syngeneic mouse model that recapitulates the successive stages of breast cancer progression and metastasis to the lungs. In this work, we used two mouse TNBC cell lines (i.e., Mvt-1 and 4T1) to generate a TNBC-MIND model in two different strains of mice. These include female FVB/N and BALB/c wild-type mice. Despite having some limitation or pitfalls such as quick progression of the disease from DICS to metastasis and needed a brief surgery, the proposed metastatic TNBC-MIND model in syngeneic mice has several selective advantages over subcutaneous or orthotopic tumor xenografts. Unlike other models, the TNBC-MIND model illustrates sequential changes from DCIS to the invasion to metastasis into the lungs via lymphatic or hematogenous dissemination within an abbreviated time span (Fig 9). To our knowledge, these two are the first TNBC-MIND models in immunocompetent mice that can be utilized to explore the mechanisms of the progression of breast cancer and provide opportunities for drug discoveries.

The injection of TNBC cells into the mammary gland ducts through the nipple provides an ideal microenvironment of a mammary gland and possibly most closely resembles a spontaneous mammary gland tumor [33, 35]. In this model, we found a DCIS-like structure in the ducts immediately (within two weeks) after the injection of Mvt-1 cells, and subsequently, the DCIS-like structures progressed to primary tumors and then invaded the surrounding ducts, lobules, and stroma. These steps of progression of TNBC in the MIND model are very similar to human breast cancer (Fig 1). Once a primary tumor has been established by the end of the second week after injection, two critical molecular episodes took place in the tumor microenvironments that promoted local invasion and metastasis to the lungs. These include loss of surrounding molecular barrier/fences (α-SMA positive myoepithelial layer) [42] of DCIS and differential expression of EMT markers (Figs 6 and 8). Since metastatic regulators Twist and Vimentin expressed scatteredly in the DCIS-like structure, we assume that these molecules do not interfere during the formation of DCIS by these cells or they might have some roles in the genesis of DCIS, which have not yet been discovered and thus further studies are warranted.

Previously, the mouse MIND model was used to study the DCIS stage, where human MCF-DCIS.COM (MCF-DCIS) cells isolated from primary human DCIS were injected into a mammary gland through the cleaved nipple of an immunodeficient mouse model. Although this contributed greatly to the development of our study design (Fig 1), the metastatic potential of these models has not been discussed [33, 34, 72]. Recently, a preclinical MIND model for breast cancer was developed. This model indicated an opportunity for translational research and an ability to study ER-α-positive breast cancer in a physiologically relevant hormone milieu [35]. This breast cancer model, which was generated in immunodeficient mice, provides evidence for the sequential progression of the disease with invasion and metastasis. However, this preclinical MIND-model was predominantly of the luminal-type breast cancer, and surprisingly, these studies found that this model was not fit for TNBC studies. Our studies, however, did indicate that the MIND model in a syngeneic mouse is equally useful to study TNBC progression and metastatic growth in lungs within a brief time.

Induction of lung metastasis in a mouse model via tail vein injection of tumor cells was established decades ago [73–75]. Since then, it has been the most widely used technique for metastasis research. This method was fast and made it easy to perform late metastatic growth in the lungs within a brief period. However, subsequent studies raised a question about whether tail vein injection mimics real-life lung metastasis [75]. The studies found that the development of lung metastasis from spontaneous or orthotropic primary tumors made no difference with lung metastasis that originated from cells injected through the tail vein [75]. However, the major limitation of a tail-vein injection is that it precludes a natural biological progression from primary tumor to distant lung metastasis. It was reported that the metastatic phenotype is more sensitive to micro-environment and native immunologic factors than the primary tumor phenotype [12]. Therefore, our TNBC-MIND model is more relevant for the study of an invasion-metastasis cascade because the disease progression from primary tumor to local invasion and then to metastatic growth to the lungs was taking place in the immunocompetent animals closely resembles the biological progression of human breast cancer, with the ability to depict the functional contribution of the immune system during metastatic progression. Additionally, during the entire duration of the study, the mice did not experience any significant loss or gain in body weight, which ensures minimum interference with physiological homeostasis.

In an era of targeted therapy, the choice of the appropriate model for TNBC research depends on the underlying hypothesis being tested. We believe that our "quick and easy" TNBC-MIND model in syngeneic mice will facilitate the development of improved breast cancer treatments.

## Supporting information

S1 Fig Mvt-1 cells were validated by detecting c-MYC expression in Mvt-1-tumors xenografts and in lung metastasis. (PDF)

S2 Fig Representative photographs of TNBC-MIND model in syngeneic mice. (PDF)

S3 Fig Representative photographs of lungs and blood vessels of different mice in TNBC-MIND model after two week of tumor cell inoculation. (PDF)

S4 Fig Measurements of tumor volume and body weight of the mice. (PDF)

S5 Fig Detection of tumor cells into the lymph nodes and blood vessels in tumor-bearing syngeneic mice. (PDF)

S6 Fig Effect on contralateral gland after Mvt-1 cell injection. (PDF)
