# Supplementary material for: MIND model for triple-negative breast cancer in syngeneic mice for quick and sequential progression analysis of lung metastasis
Source: PLoS One. 2018 May 29;13(5):e0198143. doi: 10.1371/journal.pone.0198143 (PMC5973560; doi:10.1371/journal.pone.0198143)
Supplement: S1 Fig — (PDF) [file pone.0198143.s001.pdf]

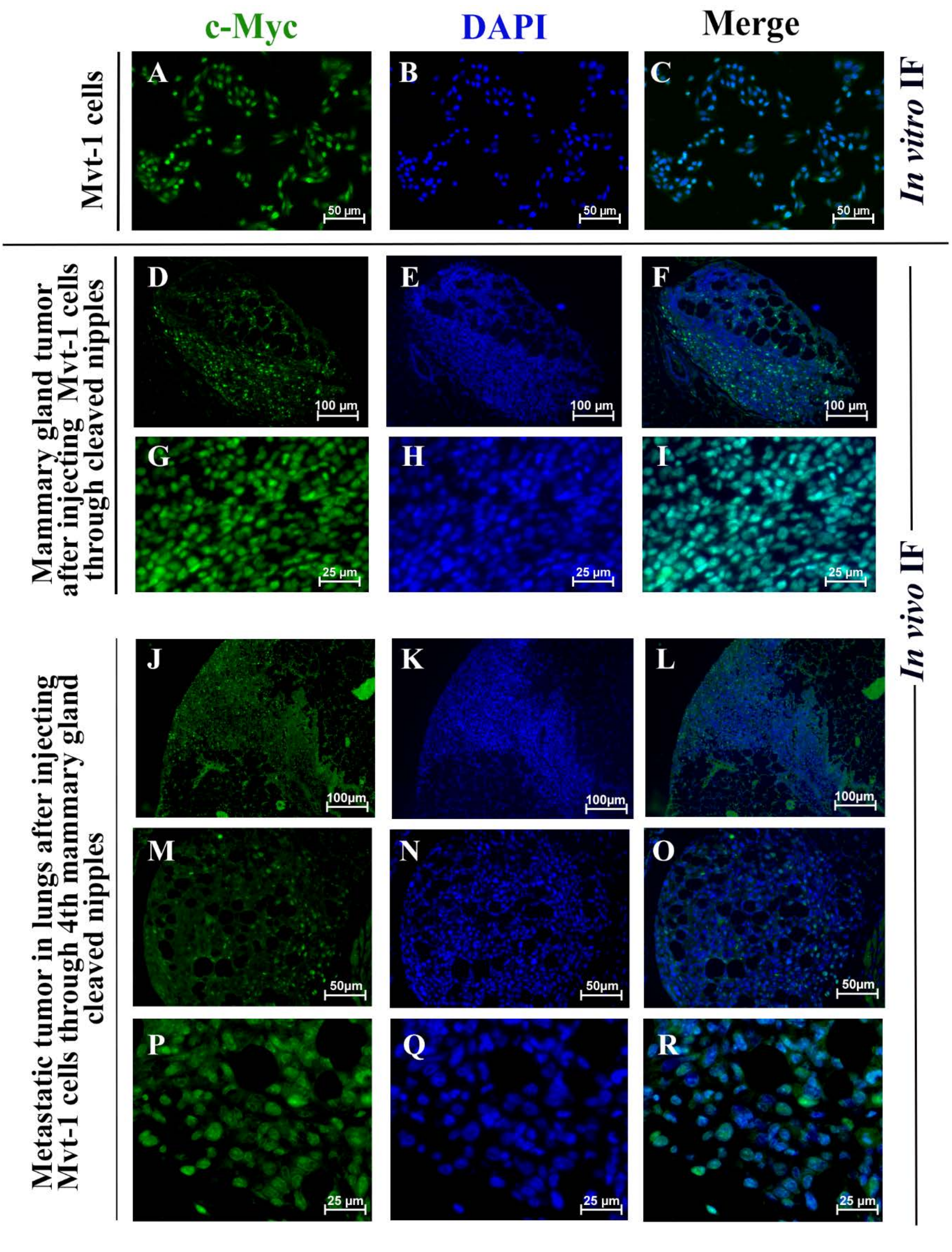

S1 Fig: Mvt-1 cells were validated by detecting c-MYC expression in Mvt-1-tumors xenografts and in lung metastasis.

(A-C): Validated Mvt-1 cell line by detecting c-MYC expression using immunofluorescence.

(D-I): Representative photographs of c-MYC expression in the tumor sections obtained from Mvt-1 primary orthotopic breast tumor xenografts in Female FVB/N mice. The scale bars represent 25-200 μm.

(J-R): Representative photographs of c-MYC expression in the sections of lung metastasis obtained from Mvt-1-orthotopic breast tumor xenografts in Female FVB/N mice. The scale bars represent 25-200 μm.
