# Supplementary material for: MIND model for triple-negative breast cancer in syngeneic mice for quick and sequential progression analysis of lung metastasis
Source: PLoS One. 2018 May 29;13(5):e0198143. doi: 10.1371/journal.pone.0198143 (PMC5973560; doi:10.1371/journal.pone.0198143)
Supplement: S2 Fig — (PDF) [file pone.0198143.s002.pdf]

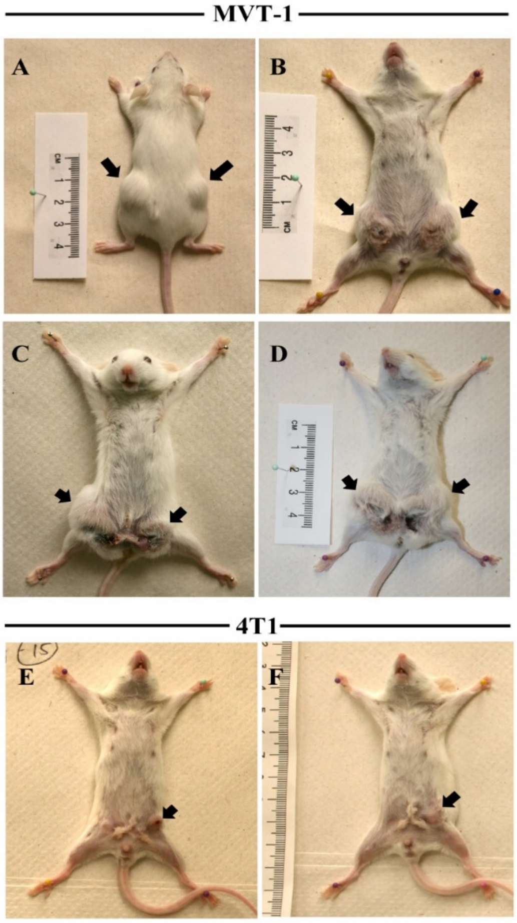

S2 Fig (related to Figure 1): Representative photographs of TNBC-MIND model in syngeneic mice.

(A-B): Representative photographs of Mvt-1-MIND tumor model in FVB/N female mice after 2-3 week of mammary intra-ductal Mvt-1 cell (10^4 cells/µl) inoculation. A, tumor visible from dorsal side, and B, tumor also visible from the ventral side.

(C-D): Representative photographs of Mvt-1-MIND tumor model in FVB/N female mice after four weeks of mammary intra-ductal MVT-1 cell injection.

(E-F): Representative photographs of 4T1-MIND tumor model in BALB/c female mice after 2nd week of mammary intra-ductal inoculation of 4T1 cell (10^4 cells/µl).
