# Supplementary material for: MIND model for triple-negative breast cancer in syngeneic mice for quick and sequential progression analysis of lung metastasis
Source: PLoS One. 2018 May 29;13(5):e0198143. doi: 10.1371/journal.pone.0198143 (PMC5973560; doi:10.1371/journal.pone.0198143)
Supplement: S3 Fig — (PDF) [file pone.0198143.s003.pdf]

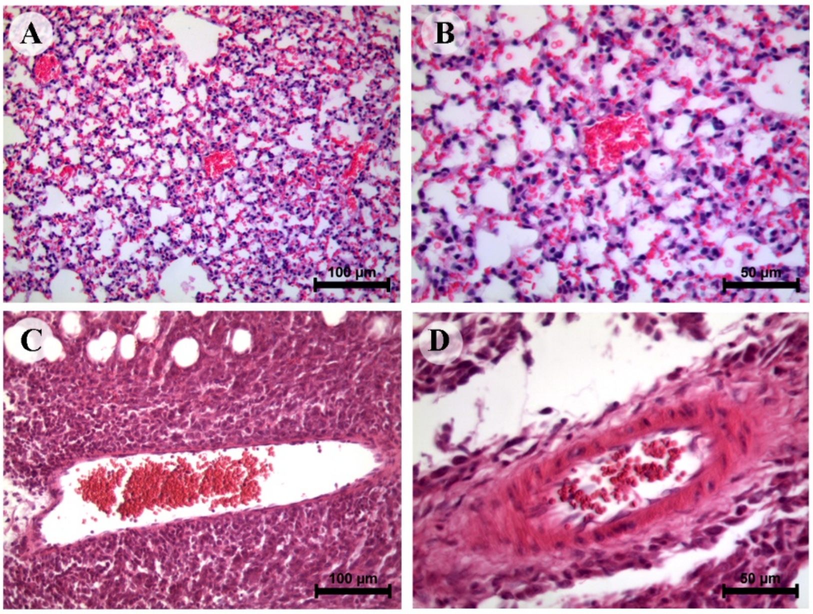

S3 Fig: (related to Figure 2): Representative photographs of lungs and blood vessels of different mice in TNBC-MIND model after two week of tumor cell inoculation.

(A-B): Tumor cells (MVT-1 or 4T1) were undetected in the lungs after two week of injection of these cells into the mammary ducts. Microscopically, lungs appeared normal. The scale bars represent 50-200 µm.

(C-D): No visible tumor cells inside the blood vessels after second week of cancer cells inoculation into the mammary ducts. The scale bars represent 50-200 µm.
