# Supplementary material for: MIND model for triple-negative breast cancer in syngeneic mice for quick and sequential progression analysis of lung metastasis
Source: PLoS One. 2018 May 29;13(5):e0198143. doi: 10.1371/journal.pone.0198143 (PMC5973560; doi:10.1371/journal.pone.0198143)
Supplement: S4 Fig — (PDF) [file pone.0198143.s004.pdf]

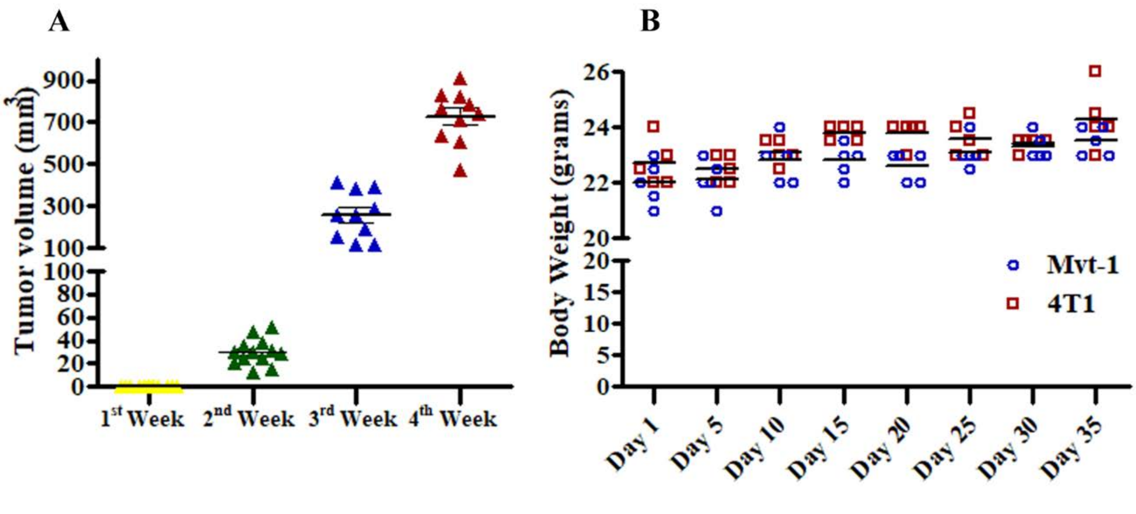

S4 Fig (Related to Figure 3): Measurements of tumor volume and body weight of the mice.

(A): Time-dependent gradual increase in tumor volume (mm^3)/gland after injection of Mvt-1cells into the 4th inguinal mammary gland duct.

(B): No significant loss or gain of body weight in mice after tumor cell inoculation for different durations.
