# Supplementary material for: MIND model for triple-negative breast cancer in syngeneic mice for quick and sequential progression analysis of lung metastasis
Source: PLoS One. 2018 May 29;13(5):e0198143. doi: 10.1371/journal.pone.0198143 (PMC5973560; doi:10.1371/journal.pone.0198143)
Supplement: S5 Fig — (PDF) [file pone.0198143.s005.pdf]

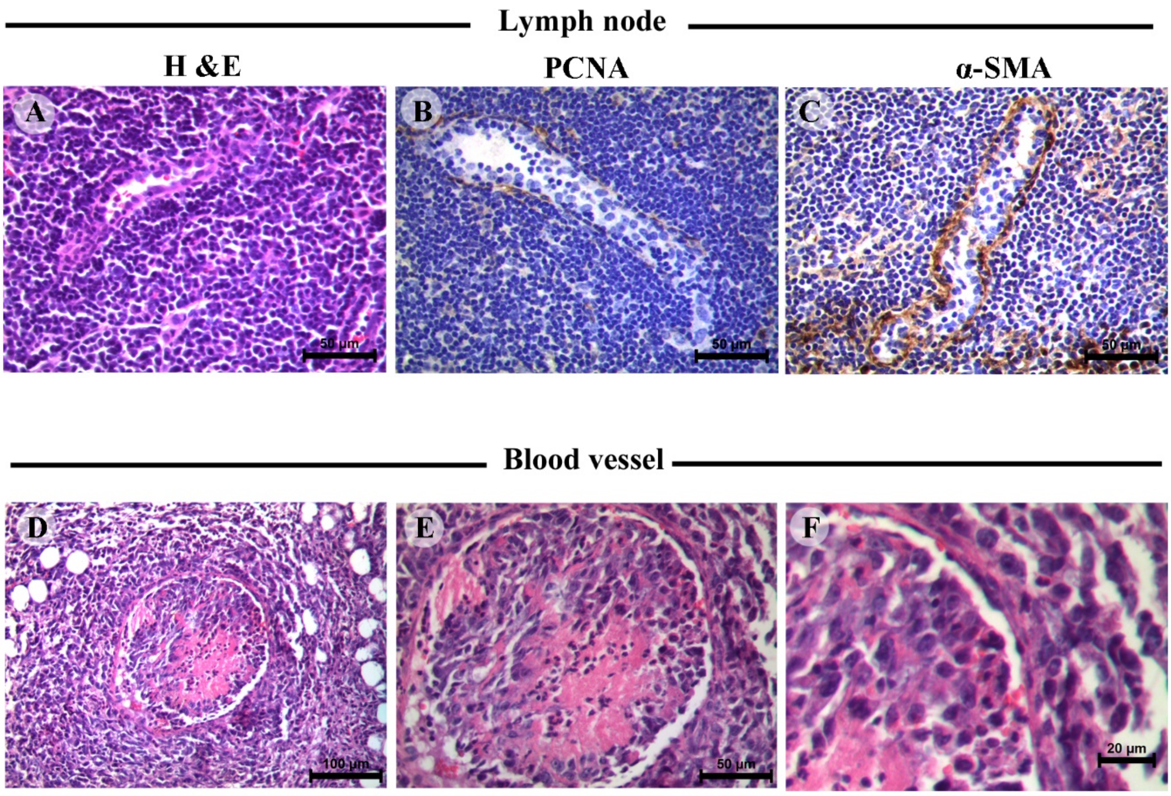

S5 Fig (Related to Figure 4): Detection of tumor cells into the lymph nodes and blood vessels in tumor-bearing syngeneic mice.

(A-C): Lymph node after 4 weeks of mammary intra-ductal injection of MVT-1 cells in the FVB/N mice. No tumor cells were found inside the lymph node. Additional staining with PCNA confirmed the absence of tumor cells inside lymph-nodes and lymphatic vessels outlined with α-SMA. The scale bars represent 50-200 µm.

(D-F): Intravasation of MVT-1 cells into the blood vessels during metastasis to secondary organ. The scale bars represent 50-200 µm.
