# Supplementary material for: MIND model for triple-negative breast cancer in syngeneic mice for quick and sequential progression analysis of lung metastasis
Source: PLoS One. 2018 May 29;13(5):e0198143. doi: 10.1371/journal.pone.0198143 (PMC5973560; doi:10.1371/journal.pone.0198143)
Supplement: S6 Fig — (PDF) [file pone.0198143.s006.pdf]

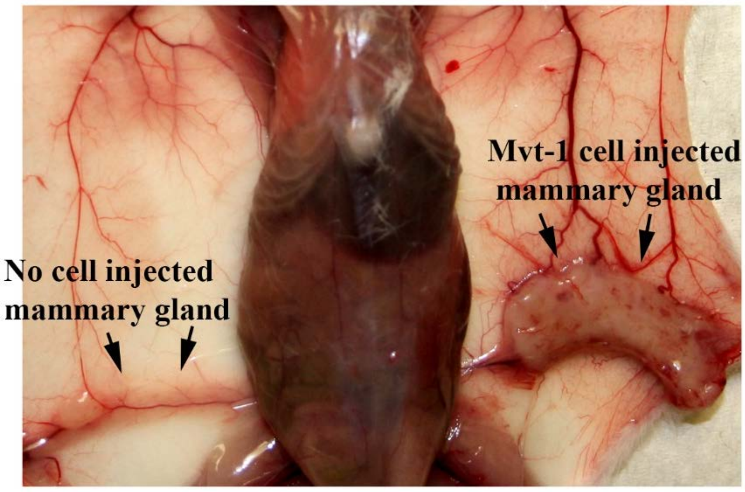

S6 Fig: Effect on contralateral gland after Mvt-1 cell injection.

Mvt-1 cells were injected on the right side 4th inguinal mammary gland of a FVB/N female mice. No changes were observed in contralateral left side mammary gland.
